# Properties of the Omicron Variant of SARS-CoV-2 Affect Public Health Measure Effectiveness in the COVID-19 Epidemic

**DOI:** 10.3390/ijerph19094930

**Published:** 2022-04-19

**Authors:** Yuki Furuse

**Affiliations:** 1 Nagasaki University Graduate School of Biomedical Sciences, 1-12-4 Sakamoto, Nagasaki 852-8523, Japan; furusey.nagasaki@gmail.com; 2 Medical Education Development Center, Nagasaki University Hospital, 1-7-1 Sakamoto, Nagasaki 852-8501, Japan

**Keywords:** COVID-19, SARS-CoV-2, variant, public health, nonpharmaceutical intervention

## Abstract

Nonpharmaceutical and pharmaceutical public health interventions are important to mitigate the coronavirus disease 2019 (COVID-19) epidemic. However, it is still unclear how the effectiveness of these interventions changes with the emergence of severe acute respiratory syndrome coronavirus 2 (SARS-CoV-2) novel variants. This simulation study utilized data from Japan and investigated how the characteristic properties of the Omicron variant, which emerged in late 2021, influence the effectiveness of public health interventions, including vaccination, the reduction of interpersonal contact, and the early isolation of infectious people. Although the short generation time of the Omicron variant increases the effectiveness of vaccination and the reduction of interpersonal contact, it decreases the effectiveness of early isolation. The latter feature may make the containment of case clusters difficult. The increase of infected children during the Omicron-dominant epidemic diminishes the effects of previously adult-targeted interventions. These findings underscore the importance of monitoring viral evolution and consequent changes in epidemiological characteristics. An assessment and adaptation of public health measures against COVID-19 are required as SARS-CoV-2 novel variants continue to emerge.

## 1. Introduction

Various public health interventions against the coronavirus disease 2019 (COVID-19) have been implemented around the world since its emergence. Nonpharmaceutical interventions still play a significant role in controlling the COVID-19 epidemic, although effective vaccines have been developed and widely used. Reducing interpersonal contact is a mainstay of such interventions. Limiting social activities by implementing lockdowns, canceling mass-gathering events, closing restaurants and bars, encouraging work from home, and closing schools can prevent the spread of the disease [1,2]. Test-trace-isolate (TTI) is another strategy to hinder viral transmission from infected persons. The TTI strategy finds infected people effectively to isolate them through extensive testing and contact tracing [3]. A cluster-based approach to identifying superspreading events also aims to detect infected people and their contacts for early isolation and quarantine [4,5].

Vaccination against severe acute respiratory syndrome coronavirus 2 (SARS-CoV-2) can prevent hospitalization and death by COVID-19 [6]. Vaccination is especially important for people at high risk of developing severe illnesses, to alleviate the burden on the healthcare system. Furthermore, vaccination can prevent viral infection itself [6]. Hence, increasing vaccination coverage forms a herd immunity that helps control the infection spread. Even if the vaccine effectiveness to prevent viral infection is not 100%, an effective reproduction number can go down in a population with a high proportion of immune people [7].

Novel variants of SARS-CoV-2 continue to emerge, imposing additional risks on public health. Some variants increase the transmissibility of the virus (e.g., Alpha and Delta), while others alter the antigenicity of the virus to escape the immunity generated by vaccination or prior infection (e.g., Beta and Gamma) [8]. The advent of the latest variant of concern, Omicron, was first reported in Botswana and South Africa in late 2021 [9]. The variant has caused a surge of cases around the world. This novel variant spreads rapidly owing to a short generation time for transmission and a substantial antigenic change [10,11,12].

Interestingly, the number of COVID-19 cases in an epidemic wave caused by the Omicron variant dramatically declined in a short period in some countries, whereas other countries continue to face issues in controlling the epidemic, as of March 2022 [13]. To understand how the properties of the Omicron variant influence the effectiveness of public health measures, this study analyzed a simulation model that reflected the COVID-19 situation in Japan with virtual scenarios of viral characteristics and public health interventions. The findings could imply the need to adapt public health measures to the novel variant.

## 2. Materials and Methods

### 2.1. Simulation Model

**Figure 1:**
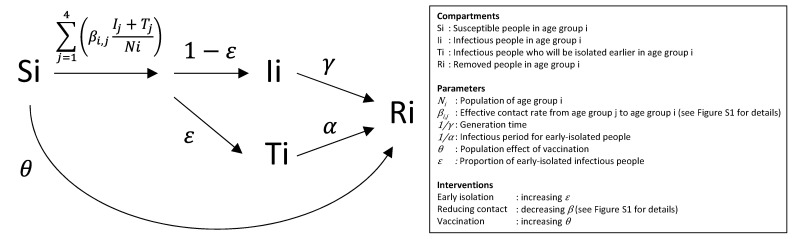
Scheme of the simulation model. The figure depicts the compartment model developed for the simulation in this study.

A deterministic compartment model was developed for the simulation in the present study, as shown in Figure 1. The compartment model comprises *S*, susceptible; *I*, infectious; *T*, infectious who will be isolated earlier by TTI; and *R*, removed. Each compartment is layered into four age groups: 0–19 years old, 20–39 years old, 40–59 years old, and ≥60 years old. The population number in each group was determined according to a demographic census in Japan [14].

*S* people get an infection from *I* and *T* people by the effective contact rate *β*. *ε*% of *I* people become isolated and lose transmissibility due to TTI at a rate *α*, and the rest (1 − *ε*)% of *I* people become *R* at a rate *γ*.

### 2.2. Infection Spread

The effective contact rate *β* was subject to change for each scenario according to the other parameters so that the total number of newly infected people increases from ~1000 to ~20,000 in 30 days in the simulation. This situation corresponds to the number of COVID-19 cases in the growing phase of the 5th and 6th epidemic waves in Japan, which were caused by the Delta variant in July–August 2021 and the Omicron variant in January–February 2022, respectively [15].

The transmission matrix among the age groups was determined according to a previous study [7]. Briefly, transmission within the same age group is four times more frequent than across different age groups, and people aged 20–59 years are four times more infectious than the other age groups (Figure S1A). The proportion of people under the age of 20 (hereafter, the age group is regarded as children) accounts for 20% of all infected people in the simulation under the transmission matrix, which is equivalent to the observation data during the Delta-dominant 5th wave of the COVID-19 epidemic in Japan [15].

### 2.3. Properties of the Omicron Variant

Three characteristic properties of the Omicron variant were explored. Firstly, the generation time of the Omicron variant is shorter than that of the Delta variant [11,12]. In the simulation model, the generation times of the Delta and Omicron variants were set as 5 and 3 days, respectively.

Secondly, the transmission matrix differs between the Delta and Omicron variants. During the Omicron-dominant 6th epidemic wave in Japan, the proportion of children among COVID-19 patients increased to ~30% [15]. This can be attributed to the efficient viral growth in the upper airway seen in this variant and a larger proportion of susceptible people in children compared to adults [7,16,17,18]. The assumption that children are 1.2 times more susceptible than adults (Figure S1A) reproduced a similar situation in the simulation model.

Thirdly, vaccination effectiveness declines for the Omicron variant [19,20]. In the simulation, it was assumed that vaccination effectiveness to prevent infection decreases by half for the Omicron variant compared with the Delta variant.

### 2.4. Interventions

Three public health interventions were investigated in the study: early isolation, the reduction of interpersonal contact, and vaccination. Early isolation by TTI increases *ε* in the simulation model (Figure 1). This increases the proportion of *T*, which has a shorter infectious period than *I*.

The reduction of interpersonal contact decreases the effective contact rate *β* in the simulation model (Figure 1). Two scenarios for contact reduction were tested: “adult-focused” and “adults and children” (Figure S1B). The intervention was assumed to work more effectively for pairs within a close age range, but little for child–adult pairs because most contact between children and adults must occur in the household [5,21]. Public health interventions on social activities seem to hardly prevent such household transmissions [21,22].

Vaccination removes a part of *S* people from the population (Figure 1). “Adult-targeted” vaccination reduces only *S* people aged ≥20, whereas “adults and children” vaccination removes the same proportion of *S* people across all age groups.

These interventions are implemented on day 30 in the simulation. The intensity of each intervention was set so that the number of newly infected people decreases to ~10,000 on day 60 in the simulation for the Delta variant. The same interventions are applied for viruses with characteristic properties of the Omicron variant in order to see the changes in the effectiveness of the interventions.

### 2.5. Data Availability

The computer script for the simulation was written in R. The model description and detailed parameter settings are available on the Github website (https://github.com/yukifuruse1217/omicron_and_measures/blob/main/SIR_japan_omicron_measures_forGit.R (accessed on 23 March 2022)).

## 3. Results

**Figure 2:**
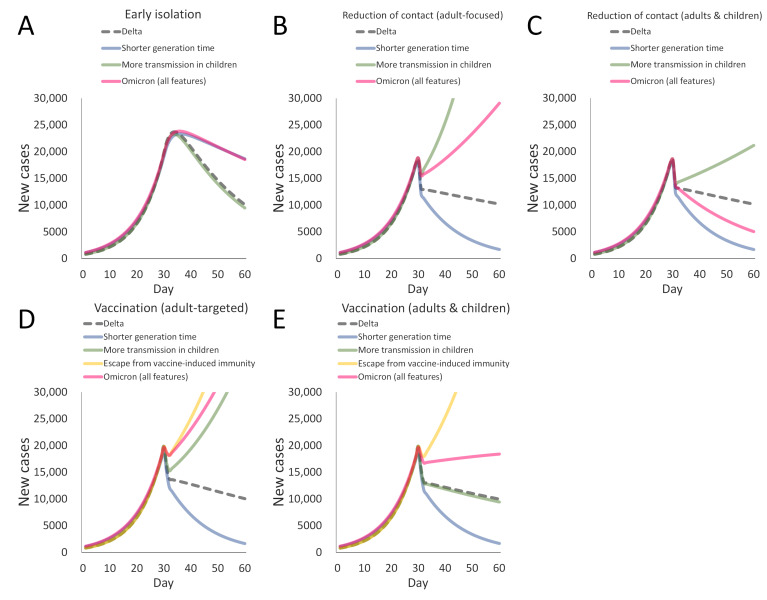
Influence of the properties of the Omicron variant on the effectiveness of public health interventions. The number of newly infected people in the 60-day simulation is shown in the figure. The infection spreads are adjusted to reproduce a similar situation for all scenarios by day 30. Thereafter, each intervention is implemented to reduce the number of new cases to the same degree for the Delta variant (black broken lines). Colored solid lines represent the number of new cases when a circulating virus acquires different properties of the Omicron variant. Analyzed interventions are (**A**) early isolation, (**B**) “adult-focused” reduction of contact, (**C**) “adults and children” reduction of contact, (**D**) “adult-targeted” vaccination, and (**E**) “adults and children” vaccination.

The COVID-19 infection spread and its control via public health interventions were simulated for the Delta variant (a black broken line in each panel of Figure 2). Additionally, the way each property of the Omicron variant influences the effectiveness of the interventions was investigated (the colored lines in Figure 2). The results showed that the effect of early isolation by TTI is reduced for the Omicron variant due to its short generation time (Figure 2A).

On the other hand, the short generation time of the Omicron variant intensifies the effect of reducing interpersonal contact. The introduction of the short generation time rapidly decreases the number of COVID-19 cases by reducing interpersonal contact compared to the Delta variant (Figure 2B,C). However, the effect of reducing contact diminishes for the Omicron variant when the intervention focuses only on interpersonal contact among adults (Figure 2B). This occurs because transmission among children contributes more to the infection spread for the Omicron variant. Conversely, the effect of reducing interpersonal contact among children (e.g., by school closure) in addition to adult-focused intervention is more evident for the Omicron variant than for the Delta variant (Figure 2C).

The short generation time enhances the effect of vaccination in suppressing infection spread (Figure 2D,E). Yet, an increase in the proportion of infected children diminishes the effect of vaccination when vaccination targets only adults (Figure 2D). Vaccination for children has been approved and recently administered in some countries [23,24,25]. Child vaccination cancels the influence of the increase of infected children on vaccination effectiveness (seen by comparing the green lines in Figure 2D,E). Still, the integrative properties of the Omicron variant, including an immune-escaping ability, decrease the effect of vaccination compared to the Delta variant (Figure 2E).

**Figure 3:**
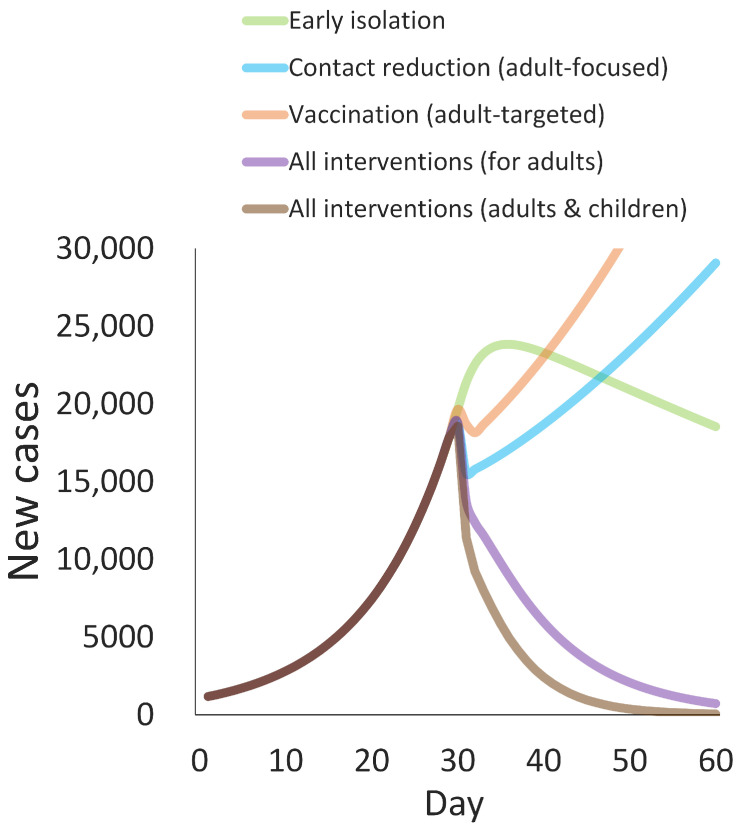
Synergistic effect of public health interventions to control the spread of the Omicron variant. Simulated epidemic curves for the Omicron variant with implementing a single public health intervention or their combination on day 30 are shown.

As already observed, the effects of early isolation, adult-focused reduction of interpersonal contact, and vaccination targeting adults decline substantially for the Omicron variant (Figure 2). However, because those interventions have different sites of action in infection spread dynamics (Figure 1), their combination can work synergistically. Implementing all the interventions has a synergistic effect on controlling the COVID-19 epidemic, even if the impact of each intervention is moderate (Figure 3). Additional public health measures for children could further help the mitigation.

## 4. Discussion

This study showed how the characteristic properties of the Omicron variant influence the effectiveness of public health interventions. Its short generation time enhances the effect of reducing interpersonal contact (e.g., by limiting social activities) and decreasing susceptible people (e.g., by vaccination). In contrast, the short generation time of the Omicron variant diminishes the effect of early isolation by TTI. The reduced impact of early isolation could cause failure in the early containment of case clusters. In fact, case clusters in preschools, schools, and nursing homes have been increasingly reported in the Omicron-dominant 6th wave of the COVID-19 epidemic in Japan [15].

Public health measures for limiting interpersonal contact are usually focused on social activities among adults. For example, closing restaurants and bars, restricting mass-gathering events, and working from home were advised during a state of emergency for COVID-19 in Japan [21,26]. Additionally, adults represent the main population for vaccine administration so far [18], as evidence regarding vaccination efficacy and safety for children was previously unavailable [27]. The proportion of infected children increased in the Omicron-dominant epidemic, possibly due to efficient viral transmission and a large proportion of non-immune people [7,16,17,18]. The effectiveness of adult-focused public health measures was shown to decline in the Omicron-dominant epidemic in this simulation study. An implementation of child-targeted public health measures, such as school closure and mass vaccination for children, might also be worth considering. However, we should prudentially discuss the pros and cons of such actions [28,29].

The model developed in this study referred to the COVID-19 situation in Japan. Still, the properties of the Omicron variant, such as the short generation time and immune-escape ability, are not unique to the country. Therefore, the reduced impact of public health interventions upon Omicron must be the case for other countries as well.

This study was not designed to measure or compare the effects of different interventions. Rather, it investigated whether the properties of the Omicron variant had a positive or negative influence on the effectiveness of public health interventions compared to the Delta variant. Models considering complex details, such as the waning of immunity, network structures, and people’s mobility, should be built to quantify the absolute impact of each public health intervention or predict the future course of the epidemic [30,31,32]. In the present simulation analysis, a single intervention with the same intensity was implemented for Delta-dominant and Omicron-dominant epidemics, and the properties of Omicron were tested one by one. Because those situations never happen in the real world, the fitness of the model cannot be discussed.

## 5. Conclusions

This study concludes that the effectiveness of public health interventions depends not only on their intensity but also on the epidemiological characteristics determined by a circulating virus. Recently, different sublineages of the Omicron variant and recombinant viral strains have emerged, and their risk assessment is underway [33]. We should keep monitoring viral evolution and consequent changes in transmission dynamics. Continuing evaluation of public health measures is important, possibly helping strategy optimization to mitigate the COVID-19 epidemic.

## Data Availability

The computer script for the simulation in this study is available on the Github website (https://github.com/yukifuruse1217/omicron_and_measures/blob/main/SIR_japan_omicron_measures_forGit.R (accessed on 23 March 2022)).

## Supplementary Materials

The following supporting information can be downloaded at: https://www.mdpi.com/article/10.3390/ijerph19094930/s1, Figure S1: Transmission matrix among the age groups.
