# Supplementary material for: Properties of the Omicron Variant of SARS-CoV-2 Affect Public Health Measure Effectiveness in the COVID-19 Epidemic
Source: Int J Environ Res Public Health. 2022 Apr 19;19(9):4930. doi: 10.3390/ijerph19094930 (PMC9099739; doi:10.3390/ijerph19094930)
Supplement: Supplementary file 1 [file ijerph-19-04930-s001.zip › ijerph-1673205-supplementary.pdf]

Supplementary material

A

Delta

To (i) \ From (j)	0–19Y (age group 1)	20–39Y (age group 2)	40–59Y (age group 3)	≥60Y (age group 4)
≥60Y (age group 4)	1	4	4	4
40–59Y (age group 2)	1	4	16	1
20–39Y (age group 2)	1	16	4	1
0–19Y (age group 1)	4	4	4	1

Omicron

To (i) \ From (j)	0–19Y (age group 1)	20–39Y (age group 2)	40–59Y (age group 3)	≥60Y (age group 4)
≥60Y (age group 4)	1	1	1	4
40–59Y (age group 2)	1	1	4	1
20–39Y (age group 2)	1	4	1	1
0–19Y (age group 1)	4.8	1.2	1.2	1.2

B

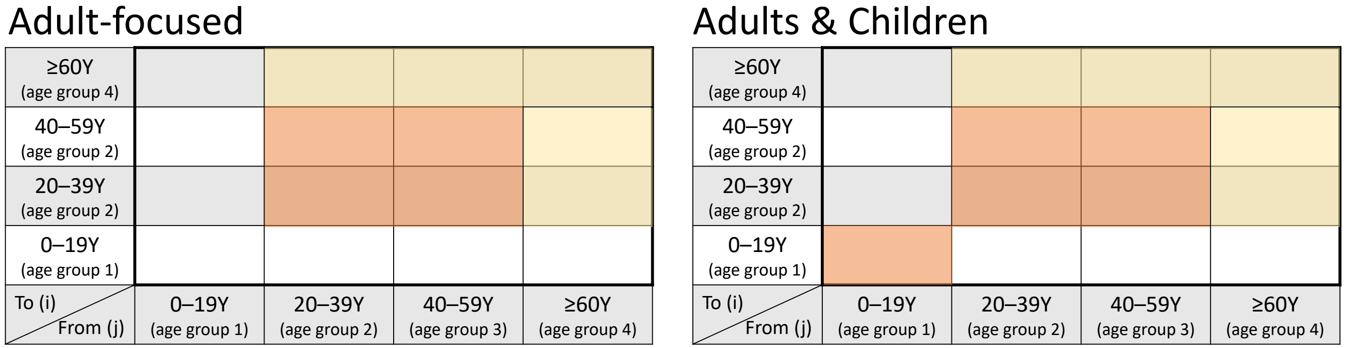

Figure S1. Transmission matrix among the age groups

A) Relative effective contact rates β between the age groups are shown. The numbers in the matrices are relative values, and they were proportionally adjusted in the simulation model (see the Materials and Methods section for details). B) The effect of reducing interpersonal contact on the transmission matrix is depicted. Orange areas indicate contact pairs reduced by the intervention, and contact pairs in yellow areas are reduced by half extent of orange areas.
